# Impact of Contact Constraints on the Dynamics of Idealized Intracranial Saccular Aneurysms

**DOI:** 10.3390/bioengineering6030077

**Published:** 2019-08-30

**Authors:** Manjurul Alam, Padmanabhan Seshaiyer

**Affiliations:** 1 Department of Bio-engineering, George Mason University, Fairfax, VA 22030, USA; 2 Department of Mathematical Sciences, George Mason University, Fairfax, VA 22030, USA

**Keywords:** intracranial aneurysms, rupture potential, nerve tissues, contact constraints, finite element analysis

## Abstract

The rupture potential of intracranial aneurysms is an important medical question for physicians. While most intracranial (brain) aneurysms are asymptomatic, the quantification of rupture potential of both symptomatic and asymptomatic lesions is an active area of research. Furthermore, an intracranial aneurysm constrained by an optic nerve tissue might be a scenario for a physician to deal with during the treatment process. In this work, we developed a computational model of an idealized intracranial saccular aneurysm constrained by a rigid nerve tissue to investigate the impact of constrained nerve tissues on the dynamics of aneurysms. A comparative parametric study for constraints of varying length on aneurysm surface was considered. Our computational results demonstrated the impact of contact constraints on the level of stress near the fundus and provided insight on when these constraints can be protective and when they can be destructive. The results show that lesions with long contact constraints generated higher stress (0.116 MPa), whereas lesions without constraints generated less stress (0.1 MPa) at the fundus, which indicated that lesions with nerve constraints can be protective and less likely to rupture than the lesions without constraints. Moreover, lesions with point load on the fundus generated the highest stress (18.15 MPa) and, hence, they can be destructive.

## 1. Introduction

Intracranial aneurysms are common phenomena in cerebral vasculature and defined as the localized dilatations of the arterial wall that mostly happen at or near bifurcations in the circle of Willis [1,2,3,4,5,6]. One of the most serious complications of intracranial aneurysms is a rupture, which results in subarachnoid hemorrhage, a disastrous occurrence in the brain with a high mortality rate of 25% to 50%. Among different types of intracranial aneurysms (such as saccular, fusiform, dissecting, mycotic), the most common type is saccular intracranial aneurysm [7]. The rupture risk of aneurysms depends on factors such as the size of the aneurysm [8], hemodynamic characteristics on the development of the aneurysm [9] and the fluid-structure interaction on the initiation, and progression of the aneurysm [10]. Although most intracranial saccular aneurysms are asymptomatic before rupture, some unruptured lesions do present symptoms when pressed against adjacent structures, such as nerve tissues [11]. An important question to be investigated is whether the rupture potential of symptomatic lesions (lesions constrained with nerve tissues) is different than that of the asymptomatic lesions (lesions without constraint) [12]. An appropriate interaction modeling is required to predict the protective or destructive role of the nerve tissue on the aneurysm. One way to model the interaction is by considering the nerve tissues as rigid contact constraints on the deformation of the aneurysm. The main purpose of this study was to build on earlier work [13,14] and test the hypothesis that a subclass of stiff contact constraints on an idealized axisymmetric intracranial saccular aneurysm can have a protective role on the aneurysm.

## 2. Materials and Methods 

The modeling of biological soft tissues, especially the mechanics of arterial walls, can be performed by the knowledge of continuum mechanics [15]. The idea of a constitutive model is important in wall mechanics to understand the mechanical responses on the arteries since those arteries are hyperelastic [16,17]. In this work, a simple idealized axisymmetric intracranial saccular aneurysm was modeled mechanically as a nonlinear hyperelastic membrane. As the most important property of hyperelastic materials is their highly nonlinear stress-strain behavior under loads, their elastic properties were different in different directions on the microstructure. Different types of constitutive modeling exist in literature to understand the mechanical response and behavior of the arterial wall [18,19,20]. Those modelings were adopted based on phenomenological relations, structure-motivated constitutive relations, and purely structural relations. The phenomenological models provided specific mathematical formulations to fit mechanical stress-strain response data. They represented the average properties of the wall tissue constituents, whereas structural models contributed the tissue microstructure elements. Exponential relations were the most prevalent among all the formulations to describe tissue response with the experimental stress-strain response. Since the modeling performed in this work was based on the phenomenological response parameters (uniaxial experimental data) of the arterial wall, a most widely used exponential Fung stress-strain relation was considered. Although the arterial wall in this work is modeled as membrane, the contribution of the tissue microstructure (elastin, collagen, and smooth muscle cell) elements due to the phenomenological nature of the Fung exponential model was considered as average properties of the wall tissue constituents [21]. The parameters used in this model were not directly related to the wall tissue microstructure and cannot be interpreted physically. Since the arterial membrane wall was responsive to the phenomenological nature of blood pressure load, the structural behavior of the tissue microstructure due to the blood cells was neglected in this work. 

The constitutive models present in two distinct formulations (strain-based and invariant-based) illustrated the mechanical responses of an anisotropic hyperelastic material [22]. The elastic response of an anisotropic hyperelastic material can be described by the governing equations of strain energy function W. In strain-based anisotropic hyperelastic modeling, the strain energy function was expressed as the components of Green strain tensors. A generalized Fung pseudostrain energy function w was used to describe the stress-strain relation of the aneurysm [23].

**Figure 1:**
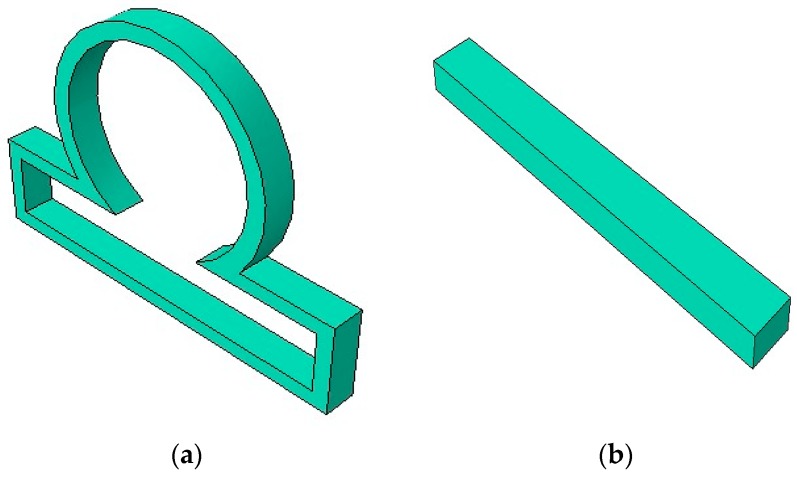
(**a**) An idealized axisymmetric intracranial saccular aneurysm model; (**b**) long rigid contact constraint.

In this work, we considered the following computational model for studying the dynamics of an intracranial aneurysm that was constrained by a contact. Figure 1 shows a section of an idealized axisymmetric intracranial saccular aneurysm model along with a long rigid contact constraint.

Governing equations are as follows:

Conservation of mass:

(1) $$J=\frac{\rho_{0}}{\rho },$$

where J=detF is the local volume ratio in the deformed state, F is the deformation gradient and ρ, $\rho_{0}$ are the mass densities of current and referential configurations respectively.

Conservation of momentum (pointwise):

(2) $$\frac{\partial t_{ij}}{\partial x_{i}}+\rho b_{j}=\rho a_{j},$$

where $b_{j}$ are the components of body force, $a_{j}$ are the components of acceleration field and $t_{ij}$ is the Cauchy stress tensor.

Cauchy stress:

(3) $$t=-pI+\frac{2}{J}F\cdot \frac{\partial W}{\partial C}\cdot F^{T},$$

where *p* is a Lagrange multiplier and $\frac{\partial W}{\partial C}$ is the second Piola-Kirchoff stress tensor.

Fung strain energy function can be written as:

(4) $$W=\frac{1}{2}c\left(e^{Q}-1\right)+\frac{1}{D}\left(\frac{1}{2}\left(J^{2}-1\right)-lnJ\right)\;\;,$$

where c and *D* are the material parameters with units of force per length.

(5) $$Q=c_{1111}E_{11}{}^{2}+c_{2222}E_{22}{}^{2}+\;c_{3333}E_{33}{}^{2}+2c_{1122}E_{11}E_{22}+\;2c_{1133}E_{11}E_{33}+\;2c_{2233}E_{22}E_{33}\;+c_{1212}\left(E_{12}{}^{2}+\;E_{21}{}^{2}\right)+\;c_{1313}\left(E_{13}{}^{2}+\;E_{31}{}^{2}\right)+\;c_{2323}\left(E_{23}{}^{2}+\;E_{32}{}^{2}\right)\;\;,$$

where $E_{ij}$. are the components of Green Lagrange strain tensors and $c_{ijkl}$. are the material parameters.

The components of Green Lagrange strain tensors $E_{ij}$ can be written as

(6) $$E_{ij}=\frac{1}{2}\left(F^{T}\cdot F-I\right)\;,$$

where *I* is the second order identity tensor.

A linear multipoint constraint requires

(7) $$A_{1}u_{i}^{P}+A_{2}u_{j}^{Q}+\ldots A_{N}u_{k}^{R}=0,$$

where $u_{i}^{P}$ is a nodal variable at node P, degree of freedom *i*, $A_{N}$ are the coefficients that define the relative motion of the nodes.

**Figure 2:**
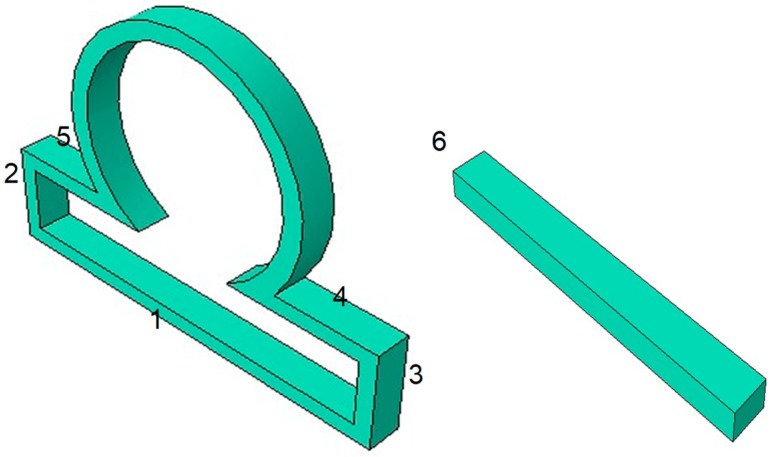
The computational geometric domain is partitioned into 6 subdomains to apply boundary conditions in each of the subdomains.

In our work, the computational domain is partitioned into the subdomains 1–6, as shown in Figure 2. Then the boundary conditions are applied on the subdomains as shown below. 

(8) $$\mathrm{Sub-domain}\;1:\;u_{\mathrm{xx}}\;=\;0,\;u_{\mathrm{xz}}\;=\;0,\;u_{\mathrm{zz}}\;=\;0,\;\frac{\partial u}{\partial n}\;=\;0,$$

(9) $$\mathrm{Sub-domain}\;2:\;u_{\mathrm{yy}}\;=\;0,\;u_{\mathrm{zz}}\;=\;0,\;u_{\mathrm{yz}}\;=\;0,\;\frac{\partial u}{\partial n}\;=\;0,$$

(10) $$\mathrm{Sub-domain}\;3:\;u_{\mathrm{yy}}\;=\;0,\;u_{\mathrm{zz}}\;=\;0,\;u_{\mathrm{yz}}\;=\;0,\;\frac{\partial u}{\partial n}=\;0,\;$$

(11) $$\mathrm{Sub-domain}\;4:\;u_{\mathrm{xx}}\;=\;0,\;u_{\mathrm{xz}}\;=\;0,\;u_{\mathrm{zz}}\;=\;0,\;\frac{\partial u}{\partial n}=\;0,\;$$

(12) $$\mathrm{Sub-domain}\;5:\;u_{\mathrm{xx}}\;=0,\;u_{\mathrm{xz}}\;=\;0,\;u_{\mathrm{zz}}\;=\;0,\;\frac{\partial u}{\partial n}=\;0,\;$$

(13) $$\mathrm{Sub-domain}\;6:\;u_{\mathrm{xx}}\;=\;0,\;u_{\mathrm{yy}}\;=\;0,\;u_{\mathrm{zz}}\;=\;0\;u_{\mathrm{xy}}\;=\;0,\;u_{\mathrm{yz}}\;=\;0,\;u_{\mathrm{xz}}\;=\;0,\;\frac{\partial u}{\partial n}=\;0,\;$$

In this work, a 3D hyperelastic anisotropic mechanical modeling of an axisymmetric intracranial saccular aneurysm was performed by using Abaqus FEA simulation software. The finite deformation modeling between the aneurysm and nerve tissue as rigid contact constraint could be performed in several ways. One way to perform interaction between a membrane and a rigid constraint was by including a weak form of contact condition via a penalty method [24]. Another useful approach was to use the idea of losing a degree of freedom when finite element node comes into contact and stays within the rigid obstacle. In this approach, as the finite element node was in contact with the constraint, it satisfied only one of the equilibrium equations along with a constraint equation for the node and then modifying the boundary conditions to solve the boundary value problem [25].

An idealized axisymmetric intracranial saccular aneurysm (ISA) was created using finite element software ABAQUS CAE, as shown in Figure 1. The created ISA had an outer radius of 18.75 mm and inner radius of 15.94 mm. The size of the aneurysm neck was 15 mm. The uniform arterial wall thickness was assumed as 2.5 mm. The lengths of the aneurysm were 60 mm and 33.75 mm in the horizontal and perpendicular directions, respectively. Once the geometry was created in ABAQUS CAE, a mesh was generated on the ISA model. The ISA wall meshed with a hexahedral element type of an 8-node, linear-brick, hybrid, constant-pressure, reduced-integration, hourglass-control (C3D8RH) material. The elements chosen for the aneurysm model were shell elements. Mesh creation of the model was performed and optimized by partitioning the model into different sections. A mesh-independent study was performed to determine the optimum number of elements. The number of elements was increased incrementally to compute the peak wall stress, and once the peak wall stress did not increase by more than 3%, then the optimum mesh size was determined. The element numbers used for the shell-type aneurysm model were 15,000. 

The length of the long contact constraint used in this work was 40 mm, whereas the length of the short contact constraint was 20 mm. The thickness of both constraints was 0.25 mm. The constraint or obstacle used in the work was considered as an isotropic elastic material with Young Modulus of 1000 MPa and Poisson’s ratio of 0.3. The element numbers used for the solid obstacle were 10,000.

Table 1 shows material constants (KPa) used in the simulation for the constitutive relation of a Fung-anisotropic model to calculate strain energy function. The values given below were determined from the pressure-diameter test, assuming the zero-stress state as the reference. To determine the material constants, experimental data were used from a previous study on the passive mechanical properties of common carotid arteries [26]. Then a series of inflation tests were performed on the blood vessels under different axial stretch ratios. Calculation of material constants was then performed from the equations that express the external force as functions of strains and material constants. Then a nonlinear least-squares method was performed to determine the material constants by optimizing the theoretical and experimental values of external force.

## 3. Results

**Figure 3:**
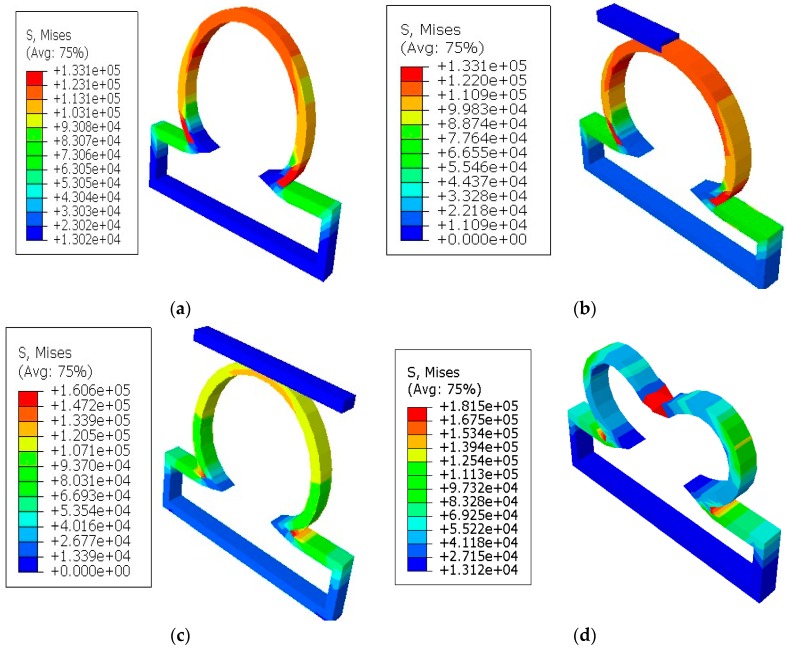

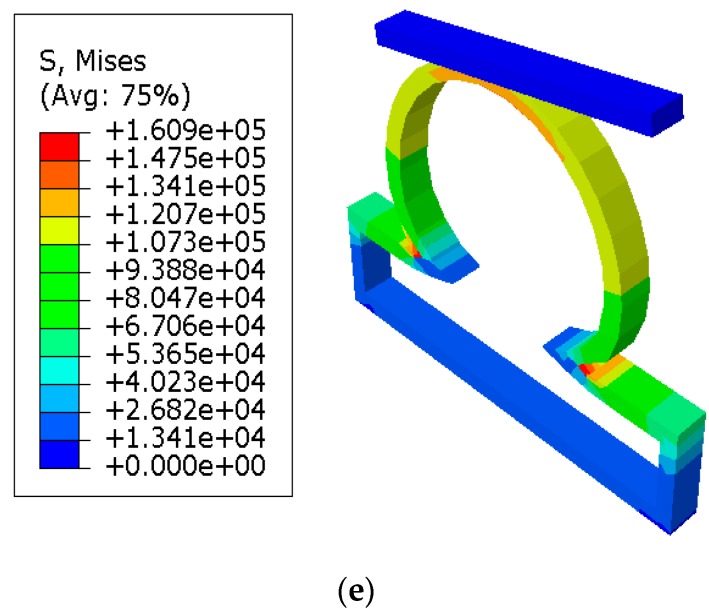
Finite element simulations of stresses (Pa) on axisymmetric intracranial saccular aneurysm for the cases where (**a**) lesions without constraints; (**b**) lesions with short contact constraints (20 mm); (**c**) lesions with long contact constraints (40 mm); (**d**) lesions with point load on the fundus; and (**e**) lesions with long contact constraints at an angle of 10 degrees from the horizontal axis. The associated stress results for all lesions were performed under the applied uniform pressure of P = 120 mmHg.

It was shown in the simulation results of Figure 3 that the multiaxial Cauchy stresses were decreased in all the lesions due to the application of short and long constraints at the lesions fundus. In this work, the neck-to-height ratio was considered greater than 1 for all lesions. The stresses at a lesion’s fundus or pole with no constraints were comparatively higher than the circumferential direction since this idealistic lesion geometry had the large radius of curvature. With the application of a large obstacle on the lesion fundus, the Cauchy stresses were decreased as the obstacle carried some of the loads from the lesion’s pole during deformation. On the other hand, lesions under short contact constraints generated slightly less stress in the fundus than the cases where lesions had no constraints. The deformed stress results shown below were under the uniform distensile pressure load (P = 120 mmHg) with their corresponding boundary conditions. It was also observed from all the results that the meridional stresses were higher than the circumferential stresses regardless of constraints in the analysis. When the lesions were under the application of point load acting on the fundus, they generated higher stresses in all directions of the lesions and could eventually lead to rupture of the lesions. Moreover, when the lesions were under the application of long constraints with an angle of 10° from the horizontal axis, they generated slightly higher stresses and were more likely to rupture than the cases where lesions were under long constraints parallel to the horizontal axis. From all the simulation results, it was observed that the stresses near the neck of the lesions were maximum.

**Figure 4:**
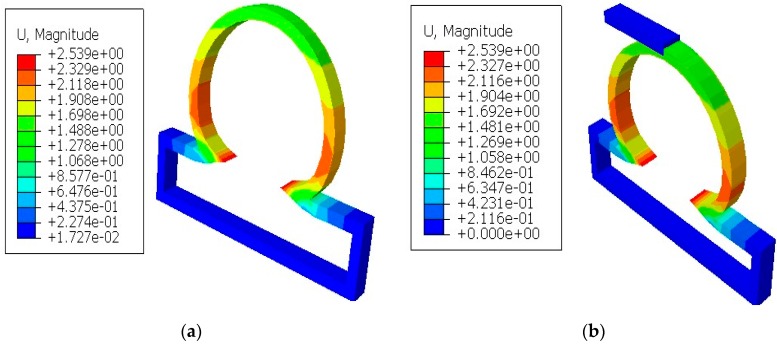

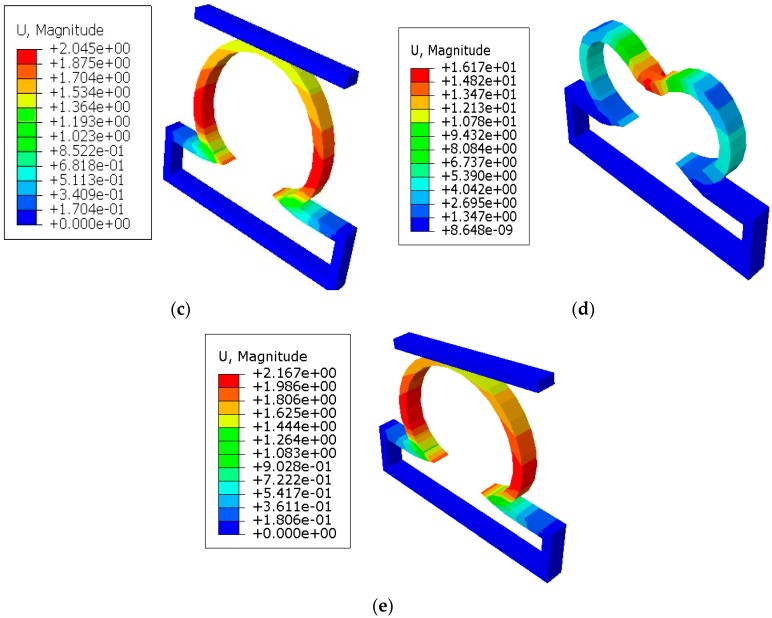
Finite element simulations of displacement (mm) on axisymmetric intracranial saccular aneurysm for the cases where (**a**) lesions without constraints; (**b**) lesions with short contact constraints (20 mm); (**c**) lesions with long contact constraints (40 mm); (**d**) lesions with point load on the fundus; and (**e**) lesions with long contact constraints at an angle of 10 degrees from the horizontal axis. The associated stress results for all lesions were performed under the applied uniform pressure of P = 120 mmHg.

In Figure 4, the displacements from the finite element simulations are shown for lesions with and without constraints. It was seen that the displacements in all lesions were greater in the circumferential direction than the meridional direction. With the application of short constraints, the displacements in lesions in the circumferential direction were increased more than the lesions with no constraints. There was a decrease in displacement seen at the lesion neck when the long constraint was applied in the aneurysm pole than in the case where the aneurysm had no constraint. When the lesions were under the application of point load, they produced higher displacements than other cases and could eventually have a higher potential to rupture.

**Figure 5:**
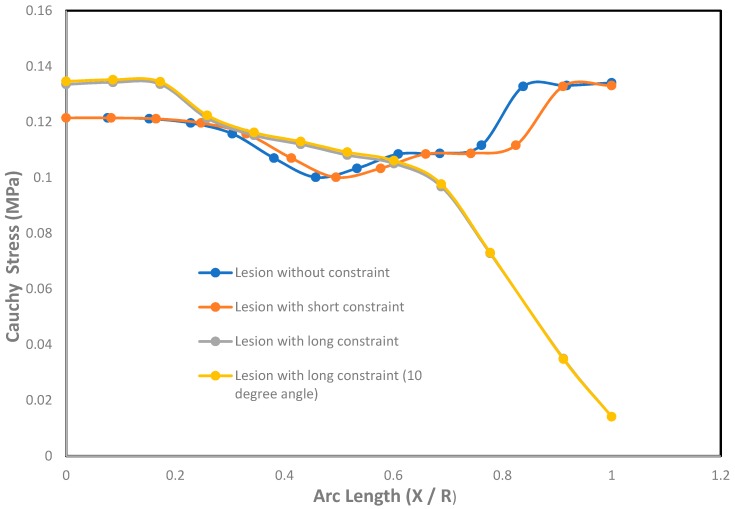
Effect of Cauchy stresses (MPa) with the undeformed arc length (X / R) on the axisymmetric intracranial saccular aneurysm for the cases where lesions without constraints, lesions with short constraints, lesions with long constraints, and lesions with long constraints at an angle of 10 degrees from the horizontal axis. Associated results were shown in both meridional and circumferential directions as a function of nondimensional undeformed arc length (X / R), where X = 0 corresponds to the fundus and X = R corresponds to the neck.

An effect of Cauchy stresses on the dimensionless undeformed arc length is shown in Figure 5. The ratio of the arc length was introduced with X and R as X ∈ [0, R], where X = 0 corresponds to the fundus or pole of the lesion and X = R corresponds to the neck of the lesion. From the graph, it was seen that the stresses on the lesions with short and long constraints were less at the pole and near the neck than the lesion without constraints. It was also seen that the stresses in the meridional direction were less than the stresses in the circumferential direction under the contact constraints. Due to the application of large contact constraints on the lesions, the stresses at the pole and near the neck were significantly decreased than the cases where the lesions had no constraints. When the lesions with long contact constraints at an angle of 10° with the horizontal axis were applied at the fundus, it was seen to generate higher stresses in both meridional and circumferential directions than the cases where long contact constraints were applied in parallel to the horizontal axis on the lesions’ fundus. This is because the lesions were in slightly imbalanced condition due to the application of long contact constraints at an angle from the horizontal axis. 

With the application of short constraints, the stresses in the lesion pole and neck were higher compared to the stresses generated in the circumferential direction, whereas long constraints produced higher stresses in the circumferential direction than the meridional directional of the lesion. 

**Figure 6:**
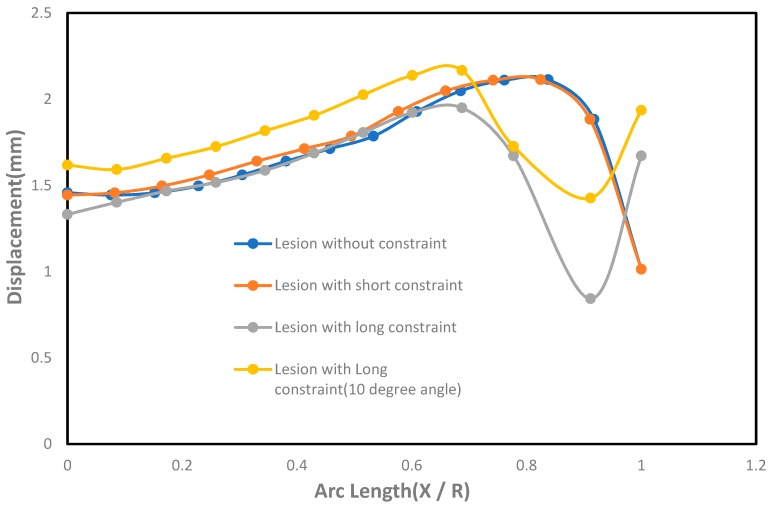
Effect of displacement (mm) with the undeformed arc length (X / R) on the axisymmetric intracranial saccular aneurysm for the cases where lesions without constraints, lesions with short constraints, lesions with long constraints, and lesions with long constraints at an angle of 10 degrees from the horizontal axis. Associated results were shown in both meridional and circumferential directions as a function of nondimensional undeformed arc length (X / R), where X = 0 corresponds to the fundus and X = R corresponds to the neck.

The effect of displacement with the undeformed arc length was shown in Figure 6. As seen in the figure, the lesions under long constraints had lower displacement near the pole and the neck than the circumferential direction compared to the cases where lesions were under no constraints. The lesions under short constraints did not have significant changes in displacement compared to the cases where lesions had no constraints. On the other hand, lesions with short constraints generated higher displacements than the lesions with long constraints, except at the lesions’ neck where long constraints generated higher displacements than the short constraints. It was also seen that the lesions under long constraints at an angle of 10° from the horizontal axis produced higher displacements in both meridional and circumferential directions than the cases where lesions were under long constraints parallel to the horizontal axis at the fundus.

## 4. Discussion

This work presents the quantification of the rupture potential of a subclass of an intracranial saccular aneurysm (ISA) under contact constraints. The hypothesis of the work: A simple, generalized axisymmetric subclass of intracranial saccular aneurysm under simple planar rigid contact constraints might be protective and might prevent the lesions from rupturing. This might be because the simple axisymmetric lesions under contact constraints generate less stress at the fundus compared to the lesions with no constraints. This hypothesis only works if we consider wall stress is the main parameter of aneurysm to be ruptured, neglecting hemodynamic force applied to the aneurysm wall, the condition of the aneurysm (i.e., size of the aneurysm, shape of the aneurysm), and types of constraints applied to the aneurysm. The simulation work was performed by a comparative parametric study varying the length of the constraints on the aneurysm pole. The results suggest that lesions under short and long contact constraints generate less meridional and circumferential stress compared to the cases where lesions had no constraints and, hence, the rupture potential of these lesions were lower compared to the ones that did not have constraints. It should be mentioned here that in our work we considered a special case of contact constraint on aneurysm fundus which works as a protective wall on the aneurysm. In this case, the contact constraint works as a balanced constraint which provides a protective effect on the aneurysm wall. This might not be the case for unbalanced constraint where the contact constraint is placed on the aneurysm laterally leading to nonuniform stress distributed throughout the aneurysm wall and eventually could work as a destructive shield for the aneurysm [27]. 

In addition to vascular mechanical wall stress, hemodynamics force or kinetic energy produced by the blood through the arteries also plays a key role in aneurysm growth and rupture. Hemodynamics force inside the arteries can be placed into three different components: (1) Hydrostatic pressure acting perpendicular to the arterial wall, (2) wall shear stress, the tangential force acting parallel to the axis of the flow direction, and (3) tensile hoop stress, the stress in the arterial wall acting in the circumferential direction due to the resulting pressure inside the arteries [28]. Since the entire fluid or blood flow characteristics inside the aneurysm were modeled here as uniform constant systolic pressure flow, wall shear stress is neglected in this work. The forces mostly acting on the aneurysm wall were hydrostatic pressure force and force due to tensile stress. We hope to address this in a forthcoming paper. The basic idea would be to include a model for the blood flow that will be described via Navier-Stokes equations, which will then be coupled to the structural Equation (2). The solution methodology that we plan to use will include solving for the velocity and pressure using open-source software such as OpenFOAM and then use that to develop the boundary conditions for the structure equations. These will solve for the new displacement for each time-step using ABAQUS and the solution will be coupled back to the Navier-Stokes and we will continue to do this iterative process until convergence. This iterative approach is an alternative to a monolithic approach where one can solve both the fluid and structure equations as a whole system [29,30,31].

It is true that a simple, axisymmetric aneurysm model cannot provide reasonable insights, but it can provide some answers about the problem overall. Moreover, it would be a good idea to do further work in this project and include more parameters (i.e., size of the aneurysm, the shape of the aneurysm, the orientation of constraints to the aneurysm, shape and size of the constraints, flow field inside the aneurysm, and extravascular fluid domain outside the aneurysm) in the simulation to get reasonable insights to this problem. Besides this, since every intracranial saccular aneurysm (ISA) is biomechanically distinct and unique, it is unrealistic to address this hypothesis for the lesion-specific model. Although this work provides some ideas about a simple, axisymmetric lesion under contact constraints, it is necessary to go further on this analysis by doing a lesion-specific model. In the analysis, the interaction between lesions and constraints was assumed frictionless contact since the aneurysms had extravascular fluid outside, which could serve as a mild lubricant. 

## Figures and Tables

**Table 1:** Material constants (KPa) for the constitutive relation of Fung-anisotropic model to calculate strain energy function.

$c_{1111}$	$c_{2222}$	$c_{3333}$	$c_{1122}$	$c_{1133}$	$c_{2233}$	$c_{1212}$	$c_{1313}$	$c_{2323}$	c	D
0.0499	1.0672	0.4775	0.0042	0.0903	0.0585	0	0	0	11.2	0.001
